# Underwater Local Cavity Welding of S460N Steel

**DOI:** 10.3390/ma13235535

**Published:** 2020-12-04

**Authors:** Jacek Tomków, Anna Janeczek, Grzegorz Rogalski, Adrian Wolski

**Affiliations:** Division of Welding Engineering, Faculty of Mechanical Engineering, Gdańsk University of Technology, Gabriel Narutowicza 11/12, 80-233 Gdańsk, Poland; anna.janeczek@pg.edu.pl (A.J.); grzegorz.rogalski@pg.edu.pl (G.R.); adrian.wolski@pg.edu.pl (A.W.)

**Keywords:** underwater welding, local cavity method, mechanical properties, gas metal arc welding, non-destructive test, destructive test, porosity

## Abstract

In this paper, a comparison of the mechanical properties of high-strength low-alloy S460N steel welded joints is presented. The welded joints were made by the gas metal arc welding (GMAW) process in the air environment and water, by the local cavity welding method. Welded joints were tested following the EN ISO 15614-1:2017 standard. After welding, the non-destructive—visual, penetrant, radiographic, and ultrasonic (phased array) tests were performed. In the next step, the destructive tests, as static tensile-, bending-, impact- metallographic (macroscopic and microscopic) tests, and Vickers HV10 measurements were made. The influence of weld porosity on the mechanical properties of the tested joints was also assessed. The performed tests showed that the tensile strength of the joints manufactured in water (567 MPa) could be similar to the air welded joint (570 MPa). The standard deviations from the measurements were—47 MPa in water and 33 MPa in the air. However, it was also stated that in the case of a complex state of stress, for example, bending, torsional and tensile stresses, the welding imperfections (e.g., pores) significantly decrease the properties of the welded joint. In areas characterized by porosity the tensile strength decreased to 503 MPa. Significant differences were observed for bending tests. During the bending of the underwater welded joint, a smaller bending angle broke the specimen than was the case during the air welded joint bending. Also, the toughness and hardness of joints obtained in both environments were different. The minimum toughness for specimens welded in water was 49 J (in the area characterized by high porosity) and in the air it was 125 J (with a standard deviation of 23 J). The hardness in the heat-affected zone (HAZ) for the underwater joint in the non-tempered area was above 400 HV10 (with a standard deviation of 37 HV10) and for the air joint below 300 HV10 (with a standard deviation of 17 HV10). The performed investigations showed the behavior of S460N steel, which is characterized by a high value of carbon equivalent (CeIIW) 0.464%, during local cavity welding.

## 1. Introduction

Underwater welding is classified as a special joining process, which is most often carried out by the wet welding method. This process is much less expensive than dry welding, which requires building special chambers [1]. The process is often carried out by shielding metal arc welding (SMAW) and flux-cored arc welding (FCAW) processes [2,3,4,5]. However, novel methods such as laser welding are studied in the latest proceedings [6]. The qualified welder should perform wet welded joints because all of the working area is surrounded by water [7]. Water increases the cooling rate, expressed by time t_8/5_ (the cooling time between temperatures 800 and 500 °C). This time is responsible for the microstructure of the heat-affected zone (HAZ) of the welded joint [8,9]. Short t_8/5_ leads to the forming of brittle microstructures in the HAZ, which is one reason for cold cracking [10]. The second negative effect generated by the water environment is high diffusible hydrogen content in the deposited metal. It can cause damages to offshore constructions [11]. Schaupp et al. [12] reported that the content of diffusible hydrogen in high-strength steels is correlated with the heat input values and cooling rate. It was assumed that a high cooling rate causes a higher hydrogen content. This effect is intensified when the welding process is transferred to the water environment. Tomków et al. [13] proved that the water environment generates about 50% more diffusible hydrogen content in deposited metal than is created during SMAW welding in the air. Klett et al. [14] reported that the amount of hydrogen increases with increasing water depth. The water depth is also responsible for the properties of underwater welded joints, which was studied by Paundra et al. [15]. It was stated that the increase of the water depth causes higher hardness in the HAZ. All these problems affect the increasing residual stresses in underwater wet welded joints, which was proved by Anggono et al. [16]. Some studies focused on improving the quality and properties of joints performed in the water environment. Tomków and Janeczek [17] proposed temper bead welding, which leads to underwater in-situ local heat treatment of high-strength low-alloy (HSLA) steel. This allows limiting the number of cold cracks. Also, Tomków et al. [18] studied the effect of waterproof coating applied on the surface of covered electrodes. Their investigations proved that hydrophobic substances could improve the quality of underwater wet welded joints. However, the joints are still characterized by the presence of cracks in the HAZ. The quality of the layers performed in a water environment can also be improved by changing the welding sequence [19]. It was stated that the welding arc stability has a strong influence on the properties of wet welded joints [20]. One of the widely tested methods, which can improve stability, is ultrasonic-assisted welding. Chen et al. [21] proved that the proposed method could improve the tensile strength of performed joints by 10%. However, it was also stated that a high-level of ultrasonic power could break the slag covering on the weld, which causes welding spatters. The ultrasonic-wave-assisted support during wet welding can lower the amount of brittle structures in the HAZ of HSLA steels, as proved by Wang et al. [22]. Wang et al. [23] also proved that ultrasonic waves could reduce the droplet diameter. It results in increasing the stability of the welding process. It was also noticed that the effect is strongly correlated with welding arc voltage. An increase of its value can generate lower quality joints. Chen et al. [24] and Shiraiwa et al. [25] presented investigations on the influence of using ultrasonic waves on the diffusible hydrogen content in the deposited metal. Both research teams stated that the proposed method could decrease the amount of hydrogen in the welded joint. However, it was also proved that the method could generate some problems, such as improper sizes of gas bubbles. This size may have a negative influence on the process stability.

Due to low cost, wet welding is the most widely used process. However, literature sources showed many limitations and problems with the quality of the joints. The number of offshore structures is increasing annually. It is necessary to find a welding method in the water environment, which allows obtaining joints with higher quality properties. These requirements resulted in the development of a local cavity welding method, in which the welding arc and the small area of the joint are isolated from the surrounding environment [26]. Rogalski et al. [27] performed bead-on-plate GMAW local cavity welding, which showed that the process could be used for cladding welds on the surface of S235JR steel. Liu et al. [28] proved that the welding speed is directly related to the forming effect and should be tested for each material. Local cavity welding was proposed for the underwater process for stainless steel. Han et al. [29] proposed metal inert gas (MIG) welding with wire located in the microdrain cover. The investigations showed that the method could be used for cladding layers on the surface of AISI 304 steel. The same material was chosen for investigations by Guo et al. [30]. The authors proposed underwater local cavity laser welding. During tests, spatters were observed in the underwater joints, typical for the laser welding process [31,32]. The testing of joints welded by local cavity welding showed that they were characterized by lower mechanical properties than joints made in the air. However, it was stated that a laser could be used in the local cavity welding of 304 stainless steel. Cui et al. [33] proposed keyhole tungsten inert gas (K-TIG) welding by the local cavity method. For testing, the S32101 duplex stainless steel was chosen. It was stated that the impact toughness values of the underwater joints reach almost 80% of the joints made in the air, which was assumed to be the proper result. Zhai et al. [34] proposed local cavity TIG welding with a flux-cored wire (FCTIG) as a method for cladding welds on the HSLA steel surface. The test showed that the maximum hardness could be observed in the HAZ near the fusion line. However, it was proved that method could be used for performing welded layers on the surface of HSLA steel in the water environment. The local cavity method was also used for performing welded joints in the water environment. Shi et al. [35] performed GMAW welding of high-strength Q690E steel produced by quenching and tempering processes. The microstructure of this material consists of tempered martensite and a small amount of bainite, which are classified as brittle microstructures. During investigations, the mechanical properties of joints welded by dry- and local dry method were compared. It was stated that the local cavity method generates more martensite in the HAZ, which results in lower mechanical properties of the tested joints. However, this test showed that local cavity welding could be used for joining 690 MPa grade steel.

HSLA steels are widely used in underwater structures [36]. The wet welding possibility of these materials has been tested, but investigations showed many problems that do not allow them to obtain high-quality joints. The literature analysis showed a lack of knowledge in the local cavity welding of the normalized HSLA steel in a water environment by the GMAW method. In this study, the comparison of the mechanical properties (tensile strength, toughness, resistance to bending, and hardness) of S460N HSLA steel welded joints is presented. This material is characterized by a very high value of carbon equivalent (CeIIW) of 0.464%. To the best of the authors’ knowledge, materials with such a high CeIIW content have not been used earlier to perform butt joints by local cavity welding. One of the joints was made in the water, and the second in the air.

## 2. Materials and Methods

### 2.1. Materials Used

For testing, S460N steel plates were chosen. Following TR ISO 15608, this grade is included in the material group 1.3. G 46 4 M21 4Si1 wire with a 1.0 mm dimension, which was chosen for welding S460N steel as a filler material. For welding, M21 gas (Ar with 18% CO_2_) was chosen to weld HSLA steels [37]. It is also a manufacturer requirement to use the mentioned gas for welding S460N steel. The chemical composition of the base material (BM) was analyzed by the spark emission spectrometry method (Hitachi, Ueden, Germany). The chemical composition and mechanical properties of the materials that were used are presented in Table 1 and Table 2.

### 2.2. Welding Process

**Figure 1:**
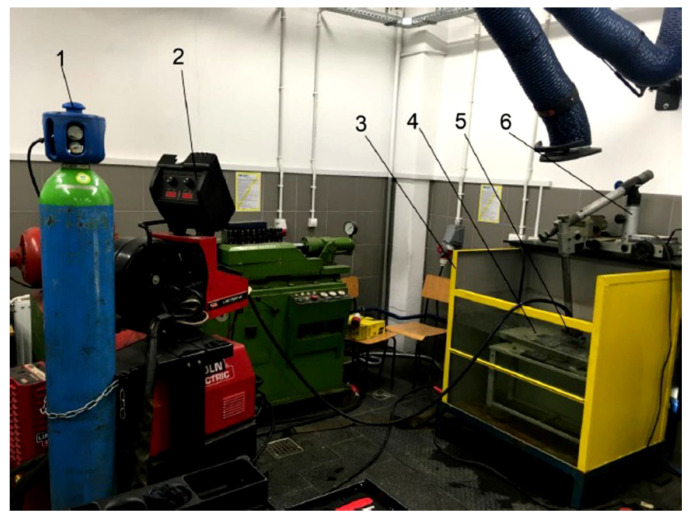
Welding test stand: 1-gas cylinder, 2-welding power source and wire feed unit, 3-tank, 4-welding table, 5-specimen, 6-welding trolley (tractor).

For testing, two specimens were welded by the automated GMAW process (Lincoln Electric, Bielawa, Poland). The first specimen was welded in the air, and the second in tap water (0.25 m depth, 20 °C), using the local cavity method. Specimens were taken in the welding position PA (1G)—welding in flat position. The welding test stand is presented in Figure 1.

**Figure 2:**
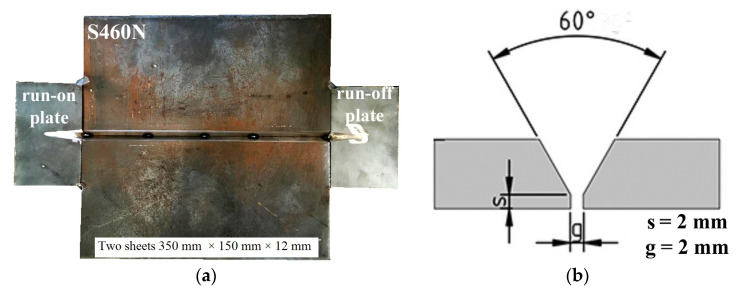
(**a**) The real view of the specimen before welding and (**b**) schematic cross-section of the specimen.

The dimensions of S460N steel plates were 350 × 150 × 12 mm, as is required in the qualification of welding procedures for butt welded joints following the EN ISO 15614-1:2017 standard [38]. Pieces of material were prepared with a “Y groove” and were joined by tack welds at the root site. The run-on and run-off plates (S460N steel, 60 × 100 × 12 mm) were used to obtain constant parameters in the tested area. The vertical view of the specimen and the schematic cross-section view are presented in Figure 2.

During preliminary investigations, bead-on-plate welds were conducted in the water to obtain welding parameters, which allow obtaining the proper weld geometry. The GMAW holder (torch) was located in the welding tractor to obtain a constant welding speed (*Vsp*). All parameters used in the experiments were in the range of parameters following the manufacturer’s requirements. For investigations, one welded joint in the water and one in the air were performed. To fulfill the welding groove, three weld passes were welded. In both environments, the same wire feed speed (*Vw*) and arc voltage (*U*) were used. In literature [8,20], HSLA steels should be welded in the water, with higher heat input (*ql*) values than during air welding. This can decrease the susceptibility to cold cracking. The investigated S460N steel is characterized by the presence of cold cracks in welded joints [39]. Due to this information, a specimen performed by local cavity welding in the water (SW) was welded with higher values of welding current (*I*) and lower *Vsp* than the specimen performed in the air (SA). Also, the gas flow (*gf*) of the shielding gas was greater for the specimens welded in the water than during air welding. After the welding of the two underwater beads, a lack of fusion was observed on the root side of the joint. This was not observed for the air welded joint. Since the welding groove was fully filled, the last weld pass (specimen SW) was cladded at the root side of the joint. After welding of the two weld passes, the specimen was turned to the other side and the third weld pass (backing weld) was performed in the position PA. The welding parameters are presented in Table 3.

### 2.3. Examination Procedure

**Figure 3:**
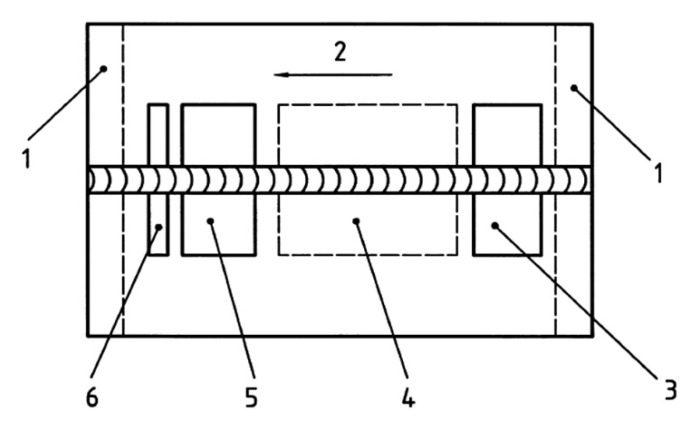
The location of test samples for destructive (DT); 1—discard 25 mm, 2—welding direction, 3—area for one tensile test specimen and bend test specimens (one for bending with tensile from the face of the weld, one for bending with tensile from the roof of the weld), 4—area for impact test specimens, 5—area for one tensile test specimen and bend test specimens (one for bending with tensile from the face of the weld, one for bending with tensile from the roof of the weld), 6—area for metallographic (macroscopic and microscopic) test specimens and one hardness test specimen.

Specimens were subjected to non-destructive (NDT) and destructive (DT) tests. In the first step, NDT was performed. The HSLA steels are characterized by high susceptibility to cold cracking in underwater conditions [8,10,20]. Cold cracks may occur up to 48 h after welding. Following this reason, the NDT was performed 48 h after welding. These kinds of tests are widely used for testing underwater constructions [40]. Both welded joints were tested using: a visual test (VT) following EN ISO 17637:2017 [41], a penetrant test (PT)—EN ISO 3452-1:2017 [42], a radiographic test (RT)—EN ISO 5579:2014 [43], and an ultrasonic test (UT), by use of an automated phased array (PA) technology—ISO 13588:2019 [44]. For RT, the ANDREX 300kV x-ray apparatus was used. During preliminary investigations, the optimal parameters were chosen: focal length 700 mm, exposure time 5 min 30 s, voltage 185 kV, anode current intensity 4.5 mA. UT was performed by PA technology with the use of a Phasor XS flaw detector (GE Research, New York, USA). For PA 16 an element head was used, with the angle of inclination at 36°. During tests, type A and S imaging were obtained. After NDT, both specimens were prepared for DT by cutting samples for each test. Investigated joints were cut following the requirements presented in the EN ISO 15614-1:2017 standard [38]. The location of test samples for DT is presented in Figure 3.

**Figure 4:**
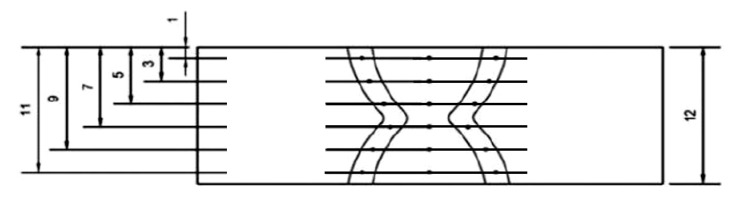
The distribution of Vickers HV10 hardness measurement points in the specimens.

At the first step, one specimen from each welded joint was subjected to a metallographic, (macroscopic and microscopic) test following the EN ISO 17639:2013 standard [45]. Samples were ground, polished, and etched by Nital 4%. In the next step, the static tensile test was performed following the requirements of the EN ISO 6892-1:2020 standard [46]. Two samples from each of the welded joints were investigated until they were broken. The maximum force was measured to calculate the tensile strength. Four samples from each welded joint were tested for the bending test (following the EN ISO 7438:2016 standard [47]). Two samples were bent with tensile at the face of the weld, and two with tensile at the root of the weld. Specimens were slightly ground to remove notches before performing tensile and three-point bending tests. During the test, the bend of the former with a diameter of ϕ = 48 mm was used (the former bend diameter for materials with elongation above 25% is four times the specimen thickness). The distance between bend rolls was 72 mm—the former bend plus double the material thickness. The specimens for the static tensile test and for the bending test were taken transversely and symmetrically to the longitudinal axis of the metal. Also, the Charpy impact test was performed for both welded joints. Prior to the Charpy impact test the specimens were etched (Nital 4%), to ensure the correct notch location. Two samples were tested from each joint with V-notch located in the HAZ and two with the V-notch located in the weld. The notch was V-shaped and has been made with an angle of 45° and a depth of 2 mm. The width and height of the sample were 10 mm. Tests were performed with requirements of the EN ISO 148-1:2017 standard [48]. The Vickers HV10 hardness was measured according to the (EN ISO 9015-1:2011 standard [49]). All tests were performed at room temperature (20 °C). The distribution of measurement points (each point in the picture means three measurements) in this test is presented in Figure 4.

## 3. Results and Discussion

### 3.1. Visual Testing

**Figure 5:**
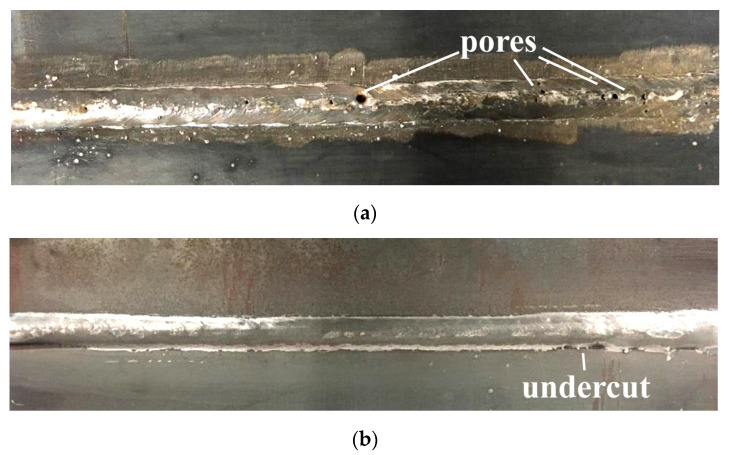
View of: (**a**) specimen SW and (**b**) specimen SA.

The views of the welded joints are presented in Figure 5. During the VT, significant differences were observed in specimens welded in both environments. In specimen SW the pores were found on the surface of the face of the weld and in the root area. These imperfections were located near the axis of the tested joint (Figure 5a). It was stated in the literature [27], that pores are the most common imperfection during local cavity GMAW welding. This statement was confirmed in the presented investigations. The surface of the joint welded in air was free of pores. However, the undercut (15 mm length) was observed on the weld face area (Figure 5b). This imperfection was observed earlier in GMAW welded joints performed with 460 grade steel [50,51].

### 3.2. Liquid Penetrant Testing

**Figure 6:**
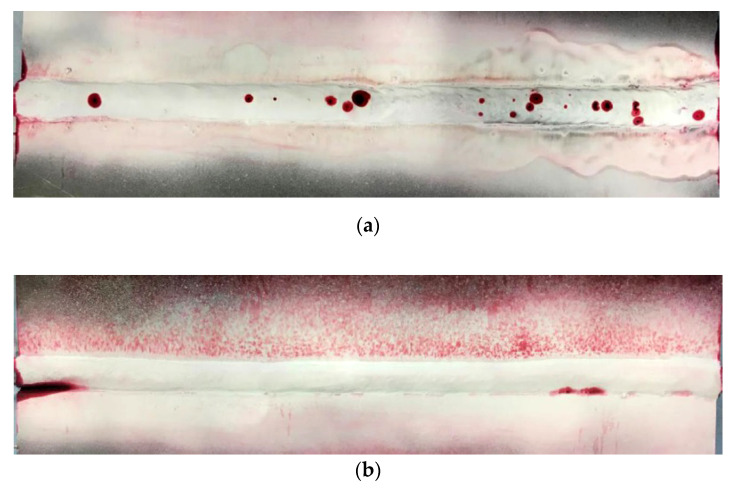
View of specimens after PT: (**a**) specimen SW and (**b**) Specimen SA.

The view of specimens after PT are presented in Figure 6. The presence of pores (specimen SW) and undercut (specimen SA) observed during VT has been confirmed in PT. Both of the mentioned tests allow observing only imperfections that occurred on the surface of the welded joints. Due to this reason, other NDT were performed.

### 3.3. Radiographic Testing

**Figure 7:**
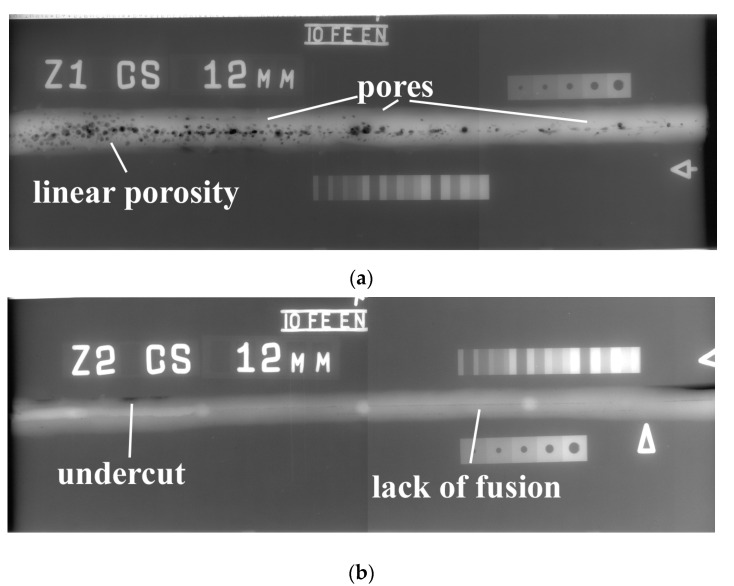
Radiograms: (**a**) specimen SW and (**b**) specimen SA.

The results of RT (radiograms) are presented in Figure 7. RT shows many differences in the quality of both tested joints. As stated in the previous investigation, S460N steel is characterized as a material with high susceptibility to the occurrence of welding imperfections [39]. The results of NDT confirms information from the literature. In the specimen welded by the local cavity method, many pores were found (Figure 7a). These imperfections occurred all along the specimen. However, the closer to the end of the joint, the lower was the number of pores. In this area, they can be characterized as linear porosity. It was stated by Nathan et al. [52] that pores can occur in HSLA steel in GMAW welding conditions. This type of imperfection is also one of the typical in underwater welding [27,53]. It resulted from the steam near the welding arc, which comes from the surrounding environment. The RT also showed that specimen SA is potentially characterized by an inside lack of fusion (80 mm). It started in ¼ length of the joint (Figure 7b). Also, the area of undercut mentioned during PT was confirmed during RT. It was stated by He et al. [54] that the preheating process can eliminate the lack of fusion defects in GMAW joints. The same process can decrease the susceptibility to cold cracking of HSLA steel. This suggests that S460N steel should be preheated before welding; this process will improve the quality of the joints.

### 3.4. Ultrasonic Testing

**Figure 8:**
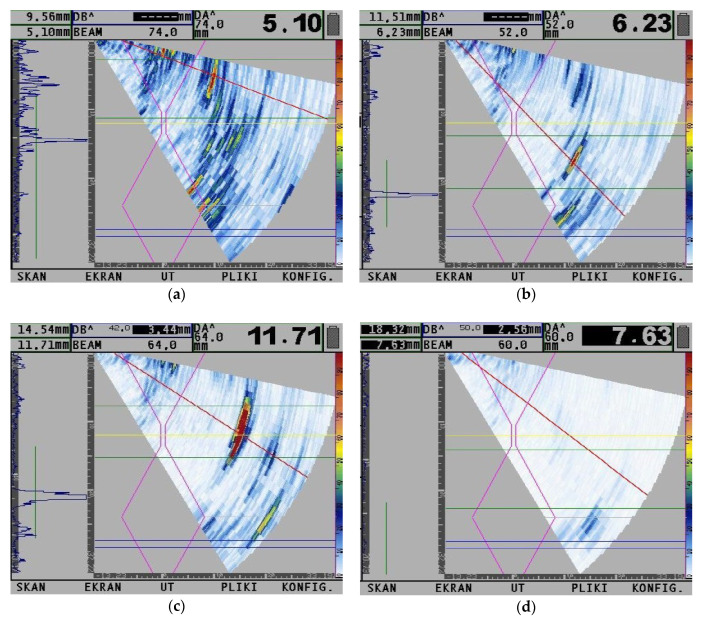
Results of the ultrasonic test (UT) by phased array (PA) technology: (**a**) beginning area of specimen SW; (**b**) finishing are of specimen SW; (**c**) lack of fusion area in specimen SA and (**d**) the area without imperfections in specimen SA.

Figure 8 presents the results of UT by PA technology. Tests were performed in areas with the highest number of indications observed during RT (specimen SW) and in the area of indication and area without any indication in the specimens welded in the air (specimen SA). For a specimen welded by the local cavity method, the differences were observed near the beginning and near the end of the joint. In the beginning, the pores were located through the whole thickness of the specimen (Figure 8a). Their number was similar from beginning of the joint to the 203 mm of its length. In the rest of the joint (from 203 to 350 mm), this number decreased, and pores changed their shape to linear. This confirmed results from RT. They were located near the root area of the weld (Figure 8b). The joint welded in the air is characterized by a lack of penetration at depth 2.5–3.8 mm (Figure 8c). However, an area without any imperfection was observed in specimen SA (Figure 8d).

### 3.5. Macroscopic Testing

**Figure 9:**
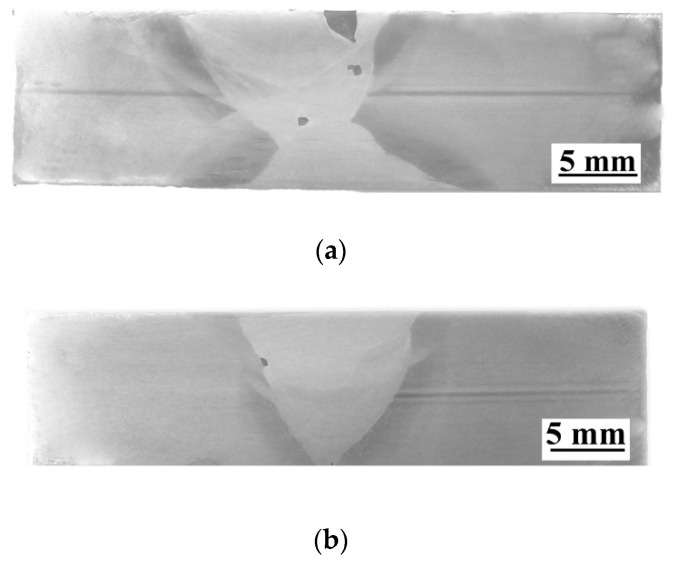
Results of macroscopic testing: (**a**) specimen SW and (**b**) specimen SA.

Macroscopic photographs are presented in Figure 9. Cross-sections in two specimens for macroscopic testing were located in areas characterized by the presence of imperfections, which were observed in NDT. Macroscopic testing of specimen SW showed one open surface pore and two pores located in the weld metal (Figure 9a). In specimen performed in the air, the pore was found in the fusion line in the area, where lack of fusion was observed during NDT. No cracks were found in both investigated joints.

### 3.6. Microscopic Testing

**Figure 10:**
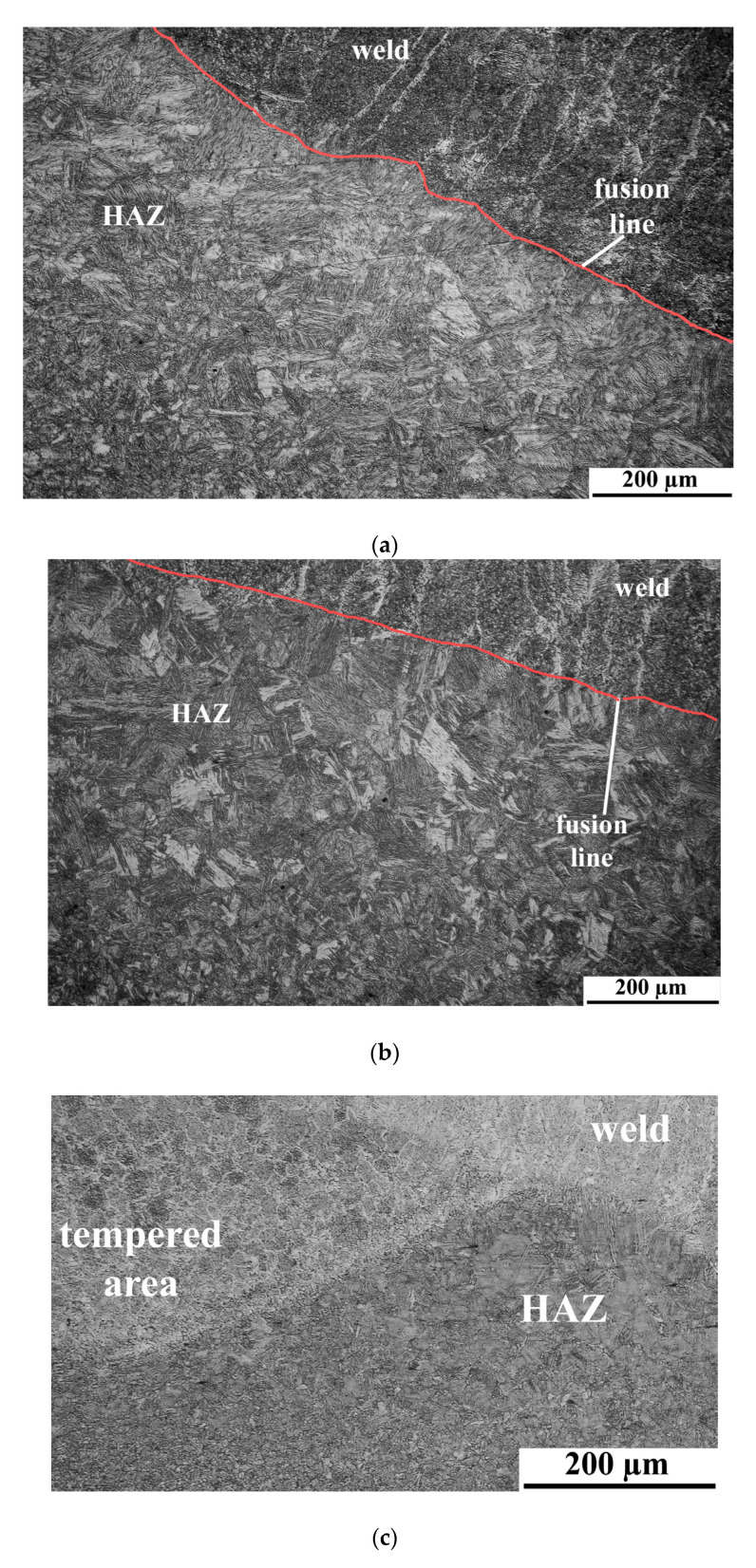

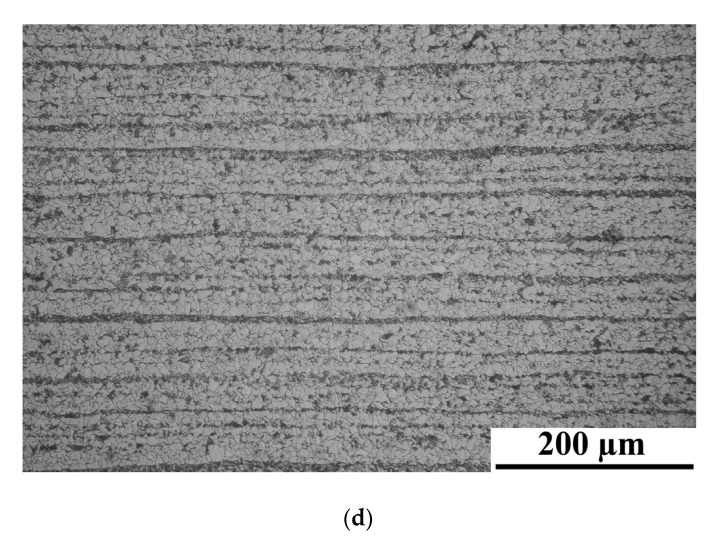
Optical micrographs of: (**a**) specimen SA; (**b**) specimen SW; (**c**) middle area of specimen SW—the tempering effect and (**d**) S460N microstructure.

The microstructures of the specimens welded in different environments are shown in Figure 10. During microscopic testing, the differences were observed for specimens welded in the air and in water. Water as a welding environment increases the cooling rate. It resulted in the formation of complex structures in the HAZ. The microstructure in HAZ of specimen SA consists of a mixture of martensite and bainite (Figure 10a). Specimen SW welded in the water is characterized by the typical structures formed in this environment. The microstructure in HAZ is coarse-grained and exhibits a martensitic morphology (Figure 10b). The weld metal of both specimens was built of dendrites. The strong effect of tempering of brittle structures in HAZ has been observed in the middle of specimen SW (Figure 10c). The heat from the weld passes has accumulated in this area, which provides the tempering of microstructures. A similar effect was presented in our further investigations [18]. The results of microscopic testing of the joint (HAZ) performed on S460N steel welded in the water by the wet welding method were presented in previous investigations [39,55]. In the presented experiments [39,55], cold cracks were found in the HAZ of the joint performed in the high cooling rate conditions. However, during the microscopic testing of specimen SW, no cracks were found. This showed the advantages of local cavity welding in comparison to the wet welding process in thermal and metallurgical conditions. The microstructure of the S460N steel is composed of banded ferrite and banded pearlite (Figure 10d).

### 3.7. Static Tensile Test

**Figure 11:**
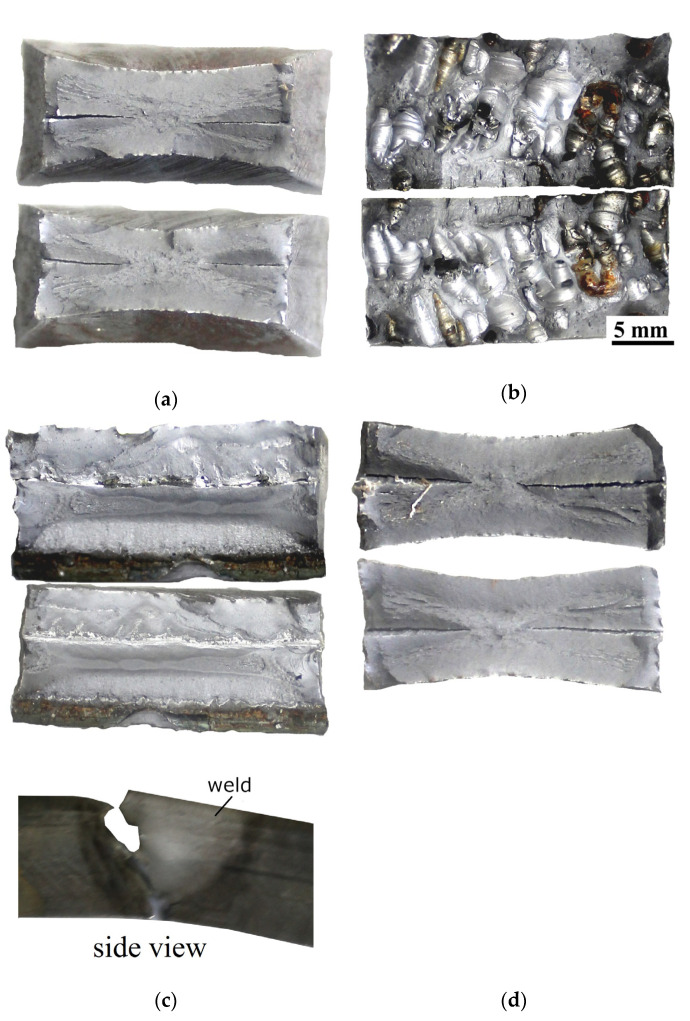
The fracture surface of the broken area during tensile testing: (**a**) Sample 1 from specimen SW, broken in base material (BM); (**b**) Sample 2 from specimen SW, broken in the weld; (**c**) Sample 1 from specimen SA, broken in the fusion line; and (**d**) Sample 2 from specimen SA, broken in the BM.

The results of tensile strength are presented in Table 4. The photographs of the fracture are presented in Figure 11. Following the EN 10025-3:2019 standard [56], the minimum tensile strength for S460N steel should be equal to 520 MPa, which is the required value for the presented investigation. From each welded joint, two samples were tested. One sample from each specimen was cut in the area near the beginning of the joint and one in the area close to the end of the specimen. Two of the samples from the joint performed by local cavity welding were cut in areas characterized by the presence of pores. During the tensile test, samples cut near the end of the joint were broken in the BM. It suggests that the number of pores did not significantly decrease tensile strength of this area (specimen SW). The broken area was characterized by plastic behavior during breaking (Figure 11a). This is common for steel BM. The second sample, which was cut near the beginning of the joint, has broken in the weld metal and did not obtain the required tensile strength (520 MPa), which was calculated as 503 MPa. The pores in this sample are located near 60% of the cross-section of the broken area (Figure 11b). One of the samples (specimen SA) was located in an area where a lack of fusion was found during NDT. During the tensile test this specimen broke at the fusion line (Figure 11c). The second sample from specimen SA was cut in the area without any observed imperfections, and it broke in the BM. The cross-section was characterized by plastic behavior during the test (Figure 11d). It was stated in the literature that tensile strength is strongly connected with the storage conditions of the filler material [57]. Different conditions may change the mechanical properties of the welded joint. However, in the presented paper, both specimens were welded with the same welding wire. This allows us to make the statement that the number of imperfections have a higher influence on the tensile strength of the welded joint than the welding environment. The tensile strength of samples from specimens welded in the water (567 MPa) and the air (570 MPa), which have broken in the BM, are characterized by a similar tensile strength value. Also, the elongation of samples without imperfections is similar. Underwater welding generates brittle microstructures such as martensite and bainite in the HAZ [27,28,29,30]. These structures are characterized by higher mechanical properties than structures generated during welding in the air. It resulted in the tensile strength of joints performed in different environments. Even the presence of many imperfections allowed us to obtain similar tensile strength for samples from different joints.

### 3.8. Bending Testing

**Figure 12:**
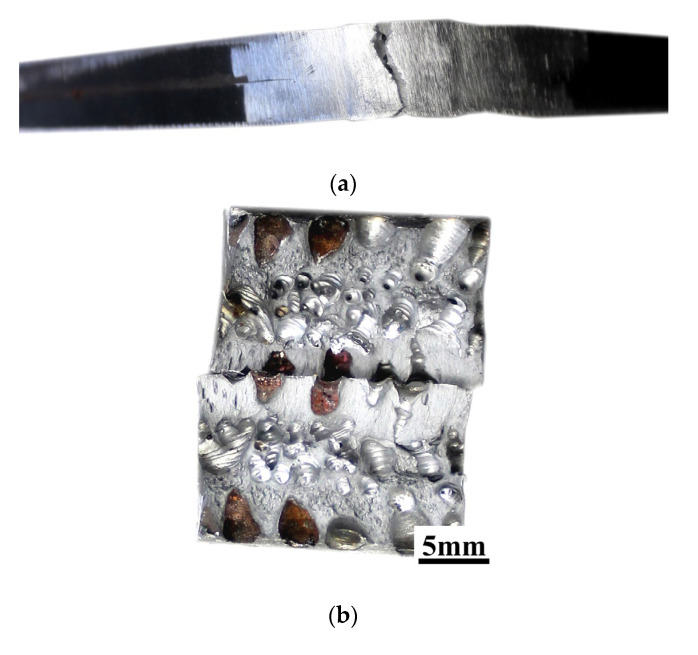

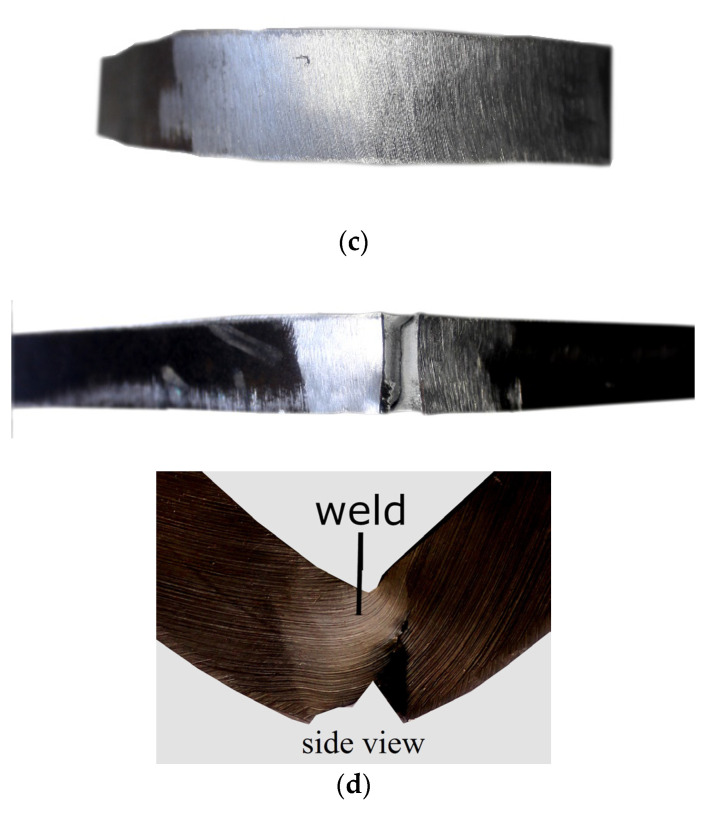
Specimens after bending test: (**a**) Sample 3 performed in the water, bent with tensile from the root of the weld; (**b**) Sample 4 performed in the water, bent with tensile from the face of the weld; (**c**) Sample 2 performed in the air, bent with tensile from the root of the weld; (**d**) Sample 3 performed in the air, bent with tensile from the root of the weld.

The results of the bending test are presented in Table 5. Samples marked by 1 and 3 were cut from the area located near the beginning of the joint. Samples marked by 2 and 4 were cut from the area near the end of the joint (following the scheme presented in Figure 3). The exemplary photographs of the bending results of the bending test are presented in Figure 12. The main aim of the bending test is to determine the plastic properties of welded joints. However, it also allows checking the influence of the welding imperfections on the properties of the welded joints, as it was stated by Rogalski et al. [58]. The criterion of acceptance was defined as a bend angle of 180°. During NDT, imperfections were found in areas, from which samples for the bending test were taken. Samples from specimen SW were broken into two parts. A maximum bending angle equal to 60° was observed for the samples bent with tensile from the root of the weld (Figure 12a), which was cut from the area close to the end of the joint. The second sample (area near the beginning of the joint), which was bent under the same conditions, broke with an angle of 10°. The cross-section observation showed that almost 60% of the weld was characterized by porosity (Figure 12b). The same was noticed for a sample bent with tensile from the face of the weld, which broke with angle 35°. The second sample (area near the end of the specimen SW) has been broken with an angle of 45°. Different results have been observed for bending the samples from the joint welded in the air. Both samples cut from the area without imperfections reached the aimed angle of 180° (Figure 12c). Specimens performed from an area characterized by the lack of fusion broke through the fusion line. The sample bent with tension from the face of the weld reached a bend angle of 110°. A sample bent with tension from the root of the weld reached bead angle 90° (Figure 12d). The results that were obtained allow for a comparison of the properties of specimens welded in both environments. The bending test results allow drawing the statement that there is no connection between tensile strength and resistance for the bending process. Both specimens could obtain similar results in the tensile test. However, there are significant differences in their behavior during bending. The cooling rate is an important factor influencing the metallurgical changes. The water environment increases the intensity of heat dissipation from the welding area in relation to the air welding conditions. It leads to formation of brittle structures in the HAZ of underwater welded joints [39,55], which was proved in this paper. Therefore, it can be assumed that the simultaneous presence of a welding imperfection and a quenched structure affects the bending test results.

### 3.9. Charpy Impact Testing

**Figure 13:**
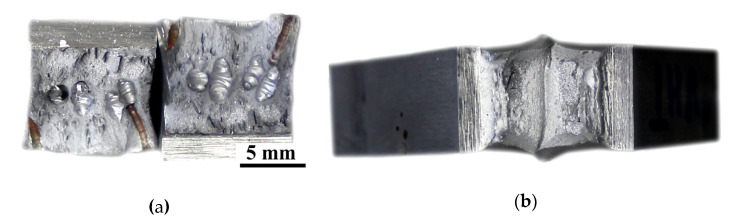
Cross-sections of samples after the Charpy impact test: (**a**) Sample 1, specimen SW, broken in the weld; and (**b**) Sample 3, specimen SA.

The results of the Charpy impact test are presented in Table 6. The exemplary photographs of samples after the test are presented in Figure 13. The results showed significant differences in weld metal impact strength (Samples 1 and 2). Both samples from the joint welded by the local cavity method have been broken into two parts after the impact test. They are characterized by an impact in the range of 39.2 to 54.9 J/cm^2^. Only one specimen from the joint performed in the air broke into two parts, with impact strength 113.8 J/cm^2^. The second one did not break. For samples with a V-notch located in the HAZ (Samples 3 and 4), the results were similar. One sample was made in the water, and in the air broke after the Charpy test, and one was in one piece. The fractography observation showed that the weld metal of specimen SW is characterized by high porosity (Figure 13a). Observation of samples with a V-notch located in the HAZ did not show significant differences. The cross-section showed areas with the typical mechanism of brittle and ductile fractures. It confirmed the statement from the bending test. Świerczyńska et al. [59] stated that the impact strength of the welded joint depends on the storage conditions of filler materials. In the presented paper, this factor was eliminated by using the same welding wire. It provided the same conditions for preparing the specimens. Results of mechanical properties tests allow drawing the following conclusion. In making welded joints that ensure tightness only (e.g., repair of the watercraft patting), porosity does not affect the operational properties, which was stated earlier in the literature [60]. High porosity cannot occur in the welded joints in the case of requirements related to the transfer of loads resulting from bending and torsional stresses.

### 3.10. Hardness Measurements

The investigated S460N steel is classified as a material of group 1.3 following the EN ISO 15614-1:2017 standard [38]. As it was stated in this standard, the maximum hardness in HAZ cannot exceed 380 HV10. This requirement was met only for joints welded in the air. The maximum measured hardness was 292 HV10 in the HAZ and 237 HV10 in the weld metal. For specimen SW, significant differences were observed. The following applies: for almost all measurement points located in the HAZ, the hardness cannot be bigger than the required value of 380 HV10. The maximum hardness was 502 HV10 in the HAZ and 299 HV10 in the weld metal. The hardness of S460N steel was in the range 189–205 HV10. The performed investigation proved the literature statement that underwater welded joints are characterized by higher hardness than joints welded in the air [7,8,35,53]. In both samples from the joint welded by the local cavity method, the lowest hardness in the HAZ was observed in the middle of the specimen. Tomków and Janeczek [18] stated that during underwater multilayer welding, the effect of tempering of brittle structures in HAZ could be observed. It causes a significant decrease in the HAZ hardness. In the presented investigations, the heat from the weld passes accumulated in the middle of the specimen. In this area, the tempered structures were observed. It resulted in lower hardness in this area of the specimen. The results of hardness measurements are presented in Table 7. The values which were higher than the required 380 HV10 have been indicated in bold and red marked.

## 4. Conclusions

In the paper, a comparison of the quality and mechanical properties of high-strength low-alloy S460N steel welded joints performed in the different environments is presented. The presented experiments showed significant differences in the properties of joints performed in the air and the water by the GMAW local cavity method. Specimen SW performed in underwater conditions; it was characterized by the presence of many pores in the weld metal. This was a result of two factors: First, the presence of water vapor inside the local cavity chamber. Second, as was stated by Hu et al. [61], even inside the local cavity chamber, the pressure affects the stability of the welding arc. It causes a decrease in the quality of the joint. As was investigated by Klett et al. [62], the stability of the welding arc affects the diffusible hydrogen content, which causes occurring pores in welded joints. The same was proved by Parshin et al. [63]. A decrease in the quality of the joint leads to a lowering of mechanical properties. To avoid gas pores from occurring, modification of the local dry cavity chamber with elements stabilizing the adhering to the base metals’ surface and allowing for the removal of the water from the groove area of the joint are required. Also, the structures formed in both environments influenced the mechanical properties. It was proved that the water environment generates structures such as coarse-grained martensite, which is characterized by high brittleness during bending and impact forces; however, this structure is characterized by high values of tensile strength. It affects the similar strength of the joint welded underwater and the air welded joint, despite the high number of gas pores formed during local cavity welding.

Obtained results allow drawing the following main conclusions:

1. The welding environment has a significant influence on the occurrence of imperfections such as gas pores in the GMAW welded joints, made by HSLA S460N steel. Joints performed by the local cavity method were characterized by many pores in the weld metal; this affected the mechanical properties. As a prospective method, the preheating process could improve the quality of the welded joint. Preheating is commonly used for welding steels with a high value of CeIIW—used S460N steel is characterized by carbon equivalent equal to 0.464%.
2. The number of imperfections have a higher influence on the tensile strength of GMAW welded joints of S460N steel than the welding environment. Samples taken from air welded specimens and samples taken from joints welded by the local cavity method are characterized by similar tensile strength—567 MPa (specimen SW) and 570 MPa (specimen SA). The high number of imperfections causes a decrease in the impact strength of joints made from S460N steel by the local cavity method from over 137.7 to 39.2 J/cm^2^. Samples characterized by high porosity broke in the weld metal.
3. The biggest differences were observed in the bending test, in which the underwater welded samples did not obtain the bending angle. It resulted in a high brittle coarse-grained martensitic microstructure that occurred in the HAZ during the welding process.
4. Specimens performed in different environments are characterized by high differences in the hardness of HAZ and the weld metal. Water highly increased the cooling rate during welding, which caused harder structures than in specimens performed in the air.
5. From the tensile strength tests carried out, it can be concluded that joints with a certain number of welding imperfections can transfer tensile stresses at the level of the base material. In a complex state of stress, such as bending, torsional, and tensile stresses, the welding imperfections (e.g., pores) significantly decrease the welded joint properties.

## Figures and Tables

**Table 1:** The chemical composition of the used materials, wt.%.

Material	C	Si	Mn	Cr	Mo	Ni	Cu	P	S	CeIIW
S460N	0.16	0.53	1.51	0.07	0.03	0.05	0.13	0.02	0.003	0.464
G 46 4 M21 4Si1 deposit	0.10	0.80	1.28	–	–	–	0.05	0.013	0.013	–

**Table 2:** Mechanical properties of the used materials following the manufacturer’s data (certificate 3.1 accordances with EN 10204).

Material	Yield Point, R_e_ (MPa)	Tensile Strength, R_m_ (MPa)	Elongation, A_5_ (%)	Toughness, KV −20 °C (J)
S460N	511	626	27.3	61
G 46 4 M21 4Si1 deposit	525	595	26.0	90

**Table 3:** Welding parameters.

Specimen	Environment	Weld Pass	*Vw* (m/min)	*gf* (l/min)	*I* (A)	*U* (V)	*Vsp* (mm/s)	*ql* (kJ/mm)
SW	water	1	10.5	35	210	32	3.7	1.82
		2			238	32	3.9	1.95
		3			230	32	4.0	1.88
SA	air	1	10.5	25	203	32	5.1	1.27
		2			197	32	4.1	1.54
		3			216	32	5.2	1.31

**Table 4:** Mechanical properties of the used materials following the manufacturer’s data.

Specimen	Sample No.	Tensile Strength, *R_m_*, (MPa)	Elongation, *A*_5_ (%)	Place of Fracture
SW (water)	1	567	26.5	BM
	2	503	12.1	Weld
SA (air)	1	523	14.0	Fusion line
	2	570	26.7	BM

**Table 5:** Results of bending test.

Specimen	Sample No	Place of Tensile	Bending Angle (°)	Fracture
SW (water)	1	face of the weld	35	weld
	2		45	weld
	3	root of the weld	60	weld
	4		10	weld
SA (air)	1	face of the weld	110	fusion line
	2		180	no
	3	root of the weld	90	fusion line
	4		180	no

**Table 6:** Results of the Charpy impact test.

Specimen	Sample	Toughness, KV, (J)	Impact Strength, KC, (J/cm^2^)	Breaking Area
SW	1	68.7	54.9	weld
	2	49.0	39.2	weld
	3	>171.6	>137.3	none
	4	147.1	117.3	HAZ
SA	1	142.2	113.8	weld
	2	>178.5	>142.8	none
	3	>158.9	>127.0	none
	4	125.1	100.1	HAZ

**Table 7:** Results of hardness Vickers HV10 measurements.

Specimen	Sample	Distance from the Face of the Weld, (mm)	Measurement Area								
			HAZ			Weld			HAZ		
			1	2	3	1	2	3	1	2	3
SW	1	1	406	416	417	229	242	227	485	473	466
		3	384	392	381	230	233	237	418	428	433
		5	363	380	357	236	225	236	313	325	332
		7	330	333	318	260	243	245	331	341	329
		8	457	461	444	266	263	243	474	461	470
		11	470	478	480	299	281	286	502	490	482
	2	1	474	480	491	285	288	279	492	489	480
		3	450	444	432	262	251	267	414	430	421
		5	331	349	337	270	268	268	305	320	313
		7	387	379	382	267	272	260	304	309	308
		9	456	444	439	297	291	285	403	399	414
		11	475	480	492	282	299	284	487	476	488
SA	1	1	279	289	290	210	222	220	287	288	279
		3	281	271	270	211	208	217	250	256	255
		5	267	266	266	212	222	214	255	241	260
		7	276	287	278	227	221	212	278	278	279
		9	248	255	247	226	219	230	286	280	270
		11	292	281	290	227	234	227	289	290	279
	2	1	253	262	270	203	209	217	252	249	248
		3	264	251	251	215	221	207	222	250	260
		5	251	254	259	213	220	214	254	251	262
		7	263	254	259	225	223	233	255	261	262
		9	274	281	278	231	239	231	280	277	263
		11	278	271	284	237	230	227	271	289	283
